# Effects of helical centerline stent vs. straight stent placement on blood flow velocity

**DOI:** 10.3389/fmedt.2023.1196125

**Published:** 2023-06-02

**Authors:** Yutaro Kohata, Makoto Ohta, Kazuyoshi Jin, Hitomi Anzai

**Affiliations:** ^1^ Graduate School of Biomedical Engineering, Tohoku University, Sendai, Japan; ^2^ Biomedical Flow Dynamics Laboratory, The Institute of Fluid Science, Tohoku University, Sendai, Japan; ^3^ ELyTMaX UMI 3757, CNRS – Université de Lyon – Tohoku University, International Joint Unit, Tohoku University, Sendai, Miyagi, Japan

**Keywords:** helical stent, angiography images, blood flow velocities, time-intensity curve (TIC), swirling flow

## Abstract

As an approach to maintain patency in femoropopliteal stenting, a helical stent configuration was proposed, which showed improved patency in clinical trials. However, the effects of helical stent placement on the flow have not been quantitatively analyzed. The purpose of this study was to estimate flow velocities to quantify the influence of helical stent placement. Helical and straight stents were implanted in three healthy pigs, and the flow velocities were estimated using the time-intensity curve (TIC) in the angiography images. The angiographic images indicated thinning of the leading edge of the contrast medium through the helically deformed artery, which was not observed in the straight stent. The slower rise of the TIC peak in the helical stent indicated faster travel of this thinner edge. Arterial expansion due to stenting was observed in all cases, and the expansion rate varied according to location. All cases of helical stent implantation showed that velocity was maintained (55.0%–71.3% velocity retention), unlike for straight stent implantation (43.0%–68.0% velocity retention); however, no significant difference was observed.

## 1. Introduction

Endovascular intervention is increasingly used for less-invasive revascularization (1, 2) according to treatment guidelines and the development of intervention techniques. Stent placement is a major endovascular treatment for arterial stenosis that mechanically widens the stenosis to secure the flow path and restore the blood supply to the distal site. After stent implantation, the degree of revascularization can be visually confirmed by injecting a contrast medium; however, restenosis may occur in some cases. Restenosis is speculated to be caused by inflammation due to vascular damage during intervention, leading to the proliferation and migration of vascular smooth muscle cells as well as the formation of an extracellular matrix, resulting in the development of neointimal hyperplasia (3).

Rocha-Sing et al. reported that 12-month vessel patency was only 28% in femoropopliteal stenting studies approved by the U.S. Food and Drug Administration (4). Although several methods to maintain patency have been proposed, including covered and drug-eluting stents (DESs) (5–10), long-term patency remains challenging.

As a mechanical approach to maintain patency, Zeller et al. proposed a helical stent configuration to change the arterial shape for a swirling flow (9). In a study of the two-year follow-up of patients, the helical stent reduced the amount of intimal hyperplasia in the femoral artery. In an animal study of helical stent implantation, Caro et al. attributed this increased patency to changes in the flow field due to arterial deformation caused by helical stents (11). They also discussed the relationship between hemodynamic conditions and oxygen transport to the vessel wall.

Several clinical, computational fluid dynamics (CFD), and cellular experimental studies have suggested that hemodynamics are associated with changes in vessel walls (8, 12–14). Based on this hypothesis, the relationship between hemodynamics and restenosis has also been investigated. Gökgöl et al. performed individualized CFD analysis in 20 patients undergoing endovascular femoropopliteal artery intervention and showed that hemodynamic parameters may predict restenosis (15). Furthermore, in stented arteries, restenotic patients have been suggested to have restenosis-prone hemodynamics, low time-averaged wall shear stress (TAWSS), and a high oscillatory shear index (OSI) during arterial deformation associated with leg flexion. Similarly, studies of carotid restenosis have reported that exposure to low TAWSS was associated with restenosis after open surgery or endovascular intervention (16, 17). Thus, the assessment of hemodynamics immediately or after stenting may predict the prognosis and help clarify the mechanisms and triggers of restenosis. The importance of examining the flow was emphasized.

However, no study has quantitatively investigated flow fields in helical stents. Due to the lack of flow information, discussions on the effects of helical stents are largely based on inferences from physical laws and other conventional stents. Therefore, quantitative measurements of the flow conditions around a stent, such as the velocity before and after stenting, are required for further discussion of the efficiency of helical stents.

Several techniques are available for measuring flow fields, including catheter measurements, ultrasonography, and magnetic resonance imaging (MRI). Recent advances in medical imaging technology have led to the development of noninvasive flow acquisition techniques. As one approach to obtain hemodynamic information noninvasively, time-intensity curve (TIC) methods based on angiography images have also been used to quantitatively estimate the velocity (18, 19). The migration of the contrast medium is detected as a spatiotemporal change in image intensity for converting the migration length to blood flow velocity. Several algorithms are available for TIC velocity measurement, and the accuracy of each has been verified through phantom tests and animal experiments (20). For example, an error of approximately 5%–18% has been reported when measuring the flow rate in a phantom tube for calculating the velocity in terms of the TIC peak-to-peak time (21, 22). As a previous study examined the *in vitro* acquisition of the TIC near a Nitinol stent (23), the TIC method was used for the comparison of stents in animal experiments.

This study aimed to compare the flow velocities of straight and helical stents before and after stenting using the TIC method. We conducted an animal test to quantitatively measure flow speed and examine the difference between helical and straight stents. This study quantitatively examines the “effect of flow modification by the helical stent on outcomes,” which has only been discussed qualitatively so far.

## 2. Method

### 2.1. Clinical setup

This study was approved by the Safety Evaluation Department of the Fukushima Medical Device Development Support Center, Fukushima, Japan. The protocol numbers were E0000061 for Cases 1 and 2 and E0000072 for Case 3.

This preliminary study included three healthy female Zen-noh premium pigs from Japan, weighing 50–59 kg. Anesthesia was injected by intravenous catheter with thiamylal sodium (3–5 mg/kg) after intramuscular injection of midazolam (0.2 mg/kg) and medetomidine (0.04 mg/kg). Buprenorphine (0.005–0.01 mg/kg) was administered intramuscularly as a preliminary analgesic. The pigs were then placed in a supine position on the operating table and ventilated with an oxygen concentration of 40%–100% and an isoflurane concentration of 1.5%–3%. After exposing the right femoral artery through a skin incision, a 6Fr femoral access sheath was inserted with a Selsinger needle into the right femoral artery. Heparin (200–300 IU/kg) was administered as an anticoagulant solution via an intravenous catheter.

A stent diameter of 6 mm was selected based on preoperative diameter measurements using angiography. Two nitinol stents were implanted in each pig, one in the left and one in the right carotid artery (LCA and RCA respectively). In Cases 1 and 2, self-expandable DES (ELUVIA, Boston Scientific, USA) as straight stents and self-expandable bare metal stents (BMS) (BM3D, Veryan Medical, UK) were used. In Case 3, a self-expandable BMS (LIFESTENT, Bard Peripheral Vascular, USA) was used as a straight stent and BM3D as a helical stent. The length of the stent was 120 mm in all cases. The appearance of a typical straight and helical stent is shown in the Supplementary Material.

A biplane angiographic C-arm Infinix Celeve-i 8000C system (Canon Medical Systems, Tokyo, Japan) was used to acquire images before and after stent placement. A 0.035 inch guidewire was inserted into the LCA and RCA. The tip of a 5Fr diagnostic catheter was advanced into the LCA/RCA to deliver the contrast medium. For each case, several sets of angiography images (1,024  ×  1024 detector pixel matrix, from 0.109–0.249 mm/px in spatial resolution, and 15 or 30 fps in temporal resolution) were acquired for digital subtraction angiography (DSA) before and after stent placement, as summarized in the Supplementary Material. All angiography images were converted to the DICOM format. At the end of the experiment, the pigs were deeply anesthetized with isoflurane concentration of 3%–5% and sacrificed with intravenous potassium chloride.

### 2.2. Data analysis

To examine arterial deformation, arterial diameters at the proximal and middle sites of the stent were measured on angiography images for the highest TIC.

Velocity estimation was performed by applying the TIC method to the angiography images. First, regions of interest (ROI) were defined, and the intensity change was analyzed for each ROI.

To set the ROIs, all vessel areas were extracted based on intensity changes in the angiography images. Based on the vessel area, a centerline of LCA and RCA was determined by applying a thinning algorithm (“skeletonize” function from the “morphology” module in the Python library “scikit-image” (24)). The extracted centerline was smoothened with a smoothing filter (“savgol_filter” function from the “signal” module in the Python library “scipy” (25)). The TIC was checked in several ROIs of 20  ×  20 pixels along the centerline, and two ROIs were then selected to calculate the velocity. The proximal ROI was set at the position where the large noise associated with the beating heart was attenuated and the peak of the TIC could be observed, and the distal ROI was set at the point where the TIC peak began to disappear. Finally, the distance between the ROIs was calculated along the centerline and used for velocity estimation.

During the acquisition of the intensity changes, the image intensity values in the ROI were averaged over the area of the ROI to reduce noise. Several local peaks were confirmed in each of the area-averaged TICs, and a time point for each of those peaks was detected as the relative minima/maxima in the area-averaged TIC using the “argrelmax” function from the “signal” module in the Python library “scipy” (25).

Velocity was calculated from the peak shift of the TIC and the distance between the two ROIs. The distance between the two ROIs, *Δ*x (px), was calculated along the vessel centerline. The peak shift, *Δ*t (frame) was defined by subtracting the peak time at the distal ROI from that at the proximal ROI for each peak. Equation 1 was used to calculate the velocity between the two ROIs for each peak in mm/s. Moreover, all the estimated velocities of the peaks were averaged. The change in the average velocity after stent treatment was calculated as velocity retention (%) using Equation 2.

(1) $$\begin{array}{c} \begin{array}{c} \begin{array}{c} \mathrm{velocity}\;(\mathrm{mm}/s)=\frac{\Delta x}{\Delta t}\times \mathrm{image}\;\mathrm{resolution}\;(\mathrm{mm}/\mathrm{px})\times \mathrm{fps}\;(\mathrm{frame}/s)\; \end{array} \end{array} \end{array}$$

(2) $$\begin{array}{c} \begin{array}{c} \mathrm{velocity}\;\mathrm{retention}\;\mathrm{ratio}\;(\text{\%})=\frac{\mathrm{average}\;\mathrm{velocity}\;(\mathrm{after})}{\mathrm{average}\;\mathrm{velocity}\;(\mathrm{before})}\times 100 \end{array} \end{array}$$

## 3. Results

**Figure 1:**
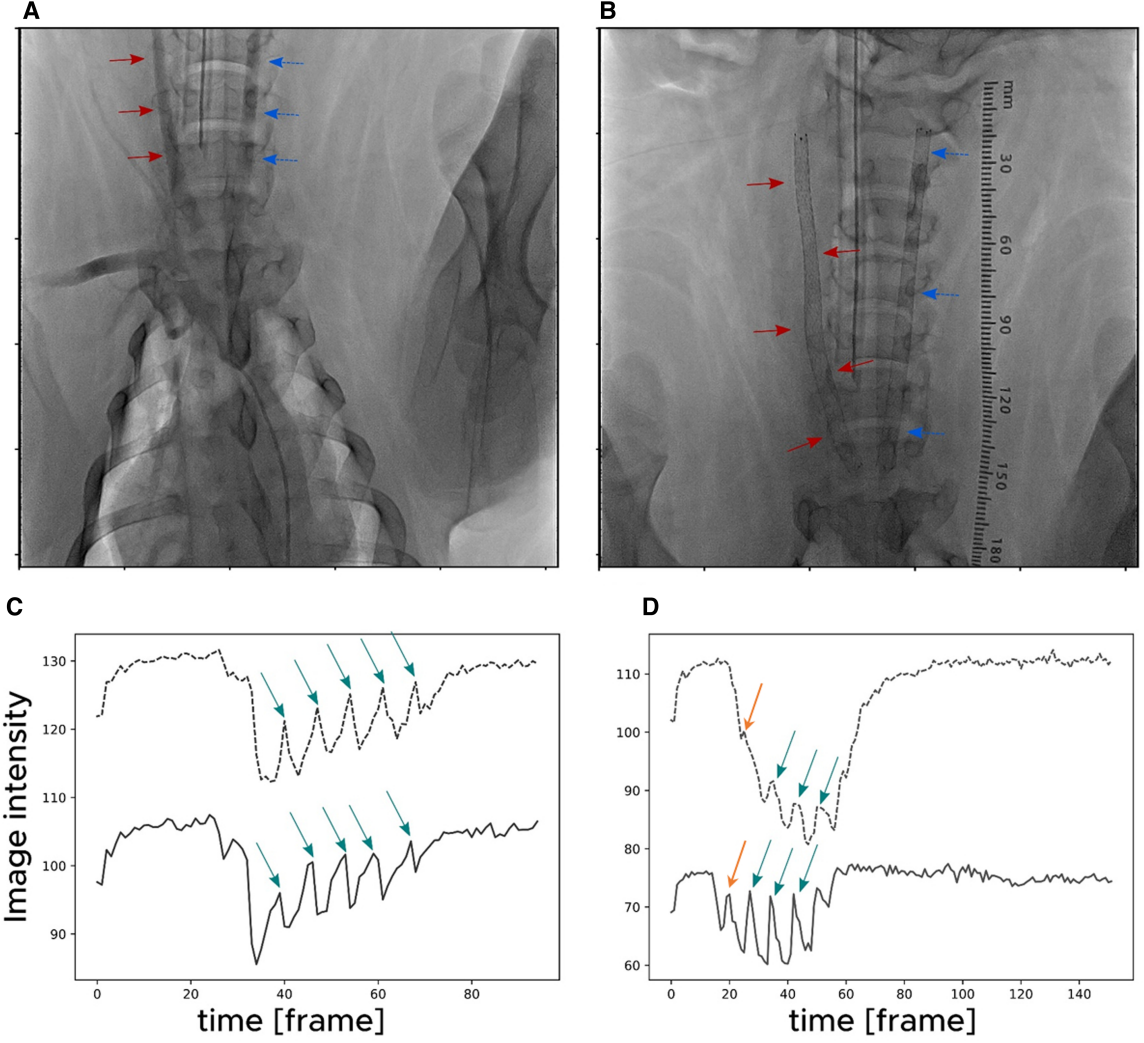
Angiography images acquired (**A**) before and (**B**) after stent placement in Case 1. The red arrows indicate the right carotid artery in which a helical stent was placed. The blue arrows indicate the left carotid artery in which a straight stent was placed. Time-intensity curves (TICs) (**C**) before and (**D**) after helical stent placement for Case 1. The solid lines and dotted lines indicate TICs at a proximal and distal region of interest (ROI), respectively. The green arrows indicate the detected peaks in the TIC.

A helical stent was implanted into the RCA, and a straight stent was implanted into the LCA in Case 1. In contrast, a helical stent was implanted into the LCA and a straight stent was implanted into the RCA in Cases 2 and 3. Figure 1 shows the angiography images and area-averaged TICs obtained before and after helical stent placement in Case 1. In the angiography images, a small torsion of the artery was observed after helical stent placement, as indicated by the red arrows in Figure 1B, which is similar to the findings of Caro et al. (11). The area-averaged TIC showed time-dependent changes in image intensity in the ROI. When the contrast medium reaches the ROI, the average intensity decreases. During several pulsations, peaks were observed in the area-averaged TIC, as indicated by the green arrow, and were detected at either 15 or 30 fps, even with the stent. By comparing the TICs between the two ROIs of the proximal and distal regions, time delays in the peaks were found. The orange arrows in Figure 1D indicate the first pulsation after stenting. The first intensity peak in Figure 1D was observed; however, the intensity change in the distal ROI was very small compared to that in the proximal ROI. The change in the intensity of the first pulsation after stenting (Figure 1D) was milder than that before stenting (Figure 1C). Therefore, the first pulse in Figure 1D was not used for the velocity estimation.

**Figure 2:**
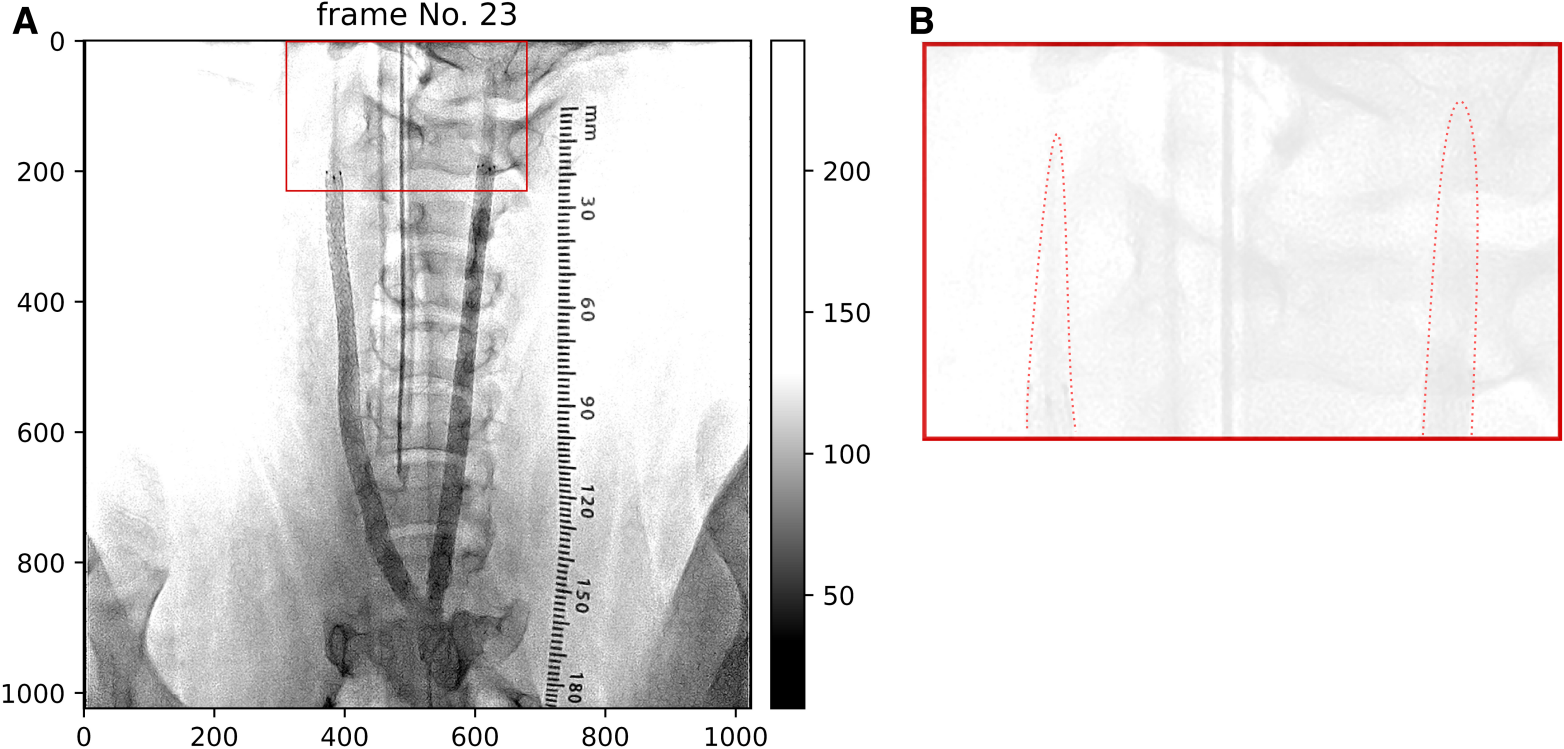
(**A**) Angiography image after stent placement in case 1. Helical deformation of the animal's right (helical-centerline stented) ICA, but not of the right (straight-centerline stented) ICA. (**B**) shows that leading edges of contrast appears thinner in helical-centerline stented ICA but not in the straight-centerline stented ICA.

Figure 2 shows the difference in the leading edges of the contrast medium between the helical- and straight-stented vessels. The image contrast was adjusted to enhance the distribution of contrast media. Figure 2 shows that there is a difference in the shape of the leading edges between the helical and straight stents; the bolus of the contrast medium seems parabolic after passing through the straight stent and thinner after passing through the helical stent.

The stent diameters are summarized in Table 1. After stent implantation, the artery expanded in all cases. The expansion rates ranged from 15.4%–49.7% for straight stents and from 10.0% – 55.0% for helical stents. The expansion rate varied at each site, suggesting that expansion was inhomogeneous along the arteries.

**Table 1:** Artery diameter before/after stent implantation. Diameter was measured at proximal and middle site of each stent, and corresponding site at before-stent artery.

			Straight		Helical	
Case			Diameter (mm)	Expansion (%)	Diameter (mm)	Expansion (%)
1	Proximal	Before	4.698	–	5.220	–
		After	5.568	18.5	5.742	10.0
	Middle	Before	4.524	–	3.480	–
		After	5.220	15.4	5.394	55.0
2	Proximal	Before	3.735	–	3.735	–
		After	5.478	46.7	4.731	26.7
	Middle	Before	3.735	–	3.486	–
		After	4.482	20.0	4.731	35.7
3	Proximal	Before	3.808	–	4.256	–
		After	4.796	25.9	5.232	22.9
	Middle	Before	3.360	–	3.584	–
		After	4.360	29.8	4.360	21.7

The estimated flow velocities for each blood vessel in each case are summarized in Table 1. Before stenting, almost no difference was observed in the average velocities of the stent arteries (327 mm/s for straight stents and 343 mm/s for helical stents). The velocity retention values are listed in Table 2. The retention ratios after helical stent placement were higher than those after straight stent placement. The retention ratios were 64.7%, 55.0%, and 71.3% for the helical stent, and 57.2%, 43.0%, and 68.0% for the straight stent.

**Table 2:** Estimated velocity (mm/s) and velocity retention ratio (%) of the arteries before and after stent placement.

Case		Straight		Helical	
		Velocity (mm/s)	Retention ratio (%)	Velocity (mm/s)	Retention ratio (%)
1	Before	441.4 ± 0.0	57.2	309.4 ± 39.8	64.7
	After	252.4 ± 29.8		200.2 ± 0.0	
2	Before	285.3 ± 19.7	43.0	452.9 ± 105.4	55.0
	After	122.7 ± 0.0		249.1 ± 42.3	
3	Before	255.6 ± 54.6	68.0	269.9 ± 43.3	71.3
	After	173.7 ± 23.2		192.5 ± 31.2	

## 4. Discussion

The velocities of helical and straight stent implantations were quantified. We applied the TIC method to angiography images of healthy pigs before and after stent implantation to estimate the velocity. The results showed that the retention ratio of helical stents was higher than that of the straight stents.

In a previous animal study by Caro et al., turning of the contrast agent after bolus injection was observed in the angiographic images of helical stents (11). They stated that this rapid movement of the contrast tip was not observed in straight stents. In the present study, no clear spirals were observed. However, the leading edge of the contrast medium was thinner, similar to that reported by Caro et al. (Figure 2). Differences in arterial geometry between helical and straight stents were also observed. Consequently, the transport of the contrast agents also changed for each stent. Faster migration of the leading edge is indicated by a slower increase in the first peak of the TIC, as shown in Figure 1. The spatial averaging of this sharp edge with the ROI resulted in a mild increase in intensity at the first pulsation in the TIC of the helical stent (Figure 1D). Therefore, helical stenting may create a flow field similar to that created by Caro et al. However, the speed of this migration has not yet been quantified.

Because contrast media easily mix with arterial blood, the contrast media transport phenomenon observed in TIC is often regarded as a fingerprint of blood flow. Various methods have been proposed to calculate velocity from the TIC, including *in vivo*, *in vitro*, animal, and clinical experiments, as summarized by Shpilfoygel et al. (20). In this study, the peak-to-peak TIC was used to estimate blood flow velocity by averaging the intensity values of the two ROIs. Thus, the passage of the contrast bolus was sensed in the high-concentration region and its spatiotemporally averaged value was calculated as the TIC velocity. A previous phantom study reported that the error rates of the TIC were 5% and 18% for steady and pulsatile flow rates, respectively (21). The TIC velocities measured in the pig carotid artery in this study ranged from 255.6 mm/s – 452.9 mm/s before stenting, which is similar to the average peak velocity measured by Doppler ultrasound in a previous study (33.3 cm/s – 46.8 cm/s) (24).

This study confirmed that the estimated velocity decreased after stent implantation in both straight and helical directions. Although blood flow retention after stenting was slightly higher with helical stents than with straight stents, the difference between straight and helical stents was not significant with respect to the SD value. The possibility of velocity reduction after stenting was also reported by Oliveira et al. in a pig aorta (26). Conversely, de Borst et al. performed stenting and velocimetry, but their results showed a reduction in diameter, resulting in a faster velocity than that before stenting (27). Table 1 shows that both straight and helical stents exhibit arterial expansion, which is similar to Caro's animal experiments (11). Based on the conservation of mass law, this expansion may have led to a decrease in velocity. In the proximal section, the velocity reduction rate tended to increase as the diameter expansion rate increased. However, in the middle section, the relationship between the rate of diameter expansion and velocity reduction weakened.

In the present study, the degree of stent expansion differed depending on position (proximal or middle), indicating that the vessel was not straight. In the present procedure, no deliberate local deformations such as pushing or pulling were performed. This inhomogeneous expansion may be partly due to the original inhomogeneous carotid arteries before stenting, as shown in Table 1. Thus, it is difficult to predict the TIC velocity retention based on diameter information alone because the internal flow distribution may have collapsed from the parabolic profile. Therefore, it is necessary to measure blood flow using measurement techniques and visually confirm expansion after surgery.

A limitation of this study is that the velocities were calculated from two ROIs on the proximal and distal sides of the angiographic images. Our peak-to-peak approach cannot generate a peak delay if the distance between the proximal and distal ROIs is insufficient; 15 fps is still higher than that in routine angiographic operations, and 15 or 30 fps is sufficient to detect a peak in this study. One or two timepoints were used to represent these time points. Therefore, higher angiographic frame rates were required to assess the velocity distribution at multiple points along the vessel.

Another limitation is that only three healthy pigs were used in this study. In addition, the presence of plaques and arterial wall heterogeneity was ignored. Based on a previous study on helical stent implantation, this study used porcine carotid arteries to observe differences in postoperative velocity retention between straight and helical stents. The results showed that both stents increased in diameter but decreased in velocity. Velocity measurements also showed that the helical stent maintained a slightly higher velocity than the straight stent. However, owing to the large SD values, it was not possible to confirm the significance between the two stents in terms of either diameter change or velocity decrease. More cases are needed in the future to confirm the influence of stents. In addition, *in vivo* and *in vitro* studies with three-dimensional reconstruction for CFD analysis are required to quantify the postoperative velocity.

In conclusion, this study used porcine carotid arteries to quantitatively evaluate the velocity changes due to stenting. The TIC velocities of helical stents were slightly higher than those of straight stents. The stent-induced expansion of the vessel may result in a decrease in velocity for both stents; however, the degree of velocity reduction was not consistent with the degree of vessel expansion in some positions. Angiographic images also suggest that more complex factors such as heterogeneous vessel expansion and helical deformation may influence velocity changes after stent implantation. Further animal studies and *in vitro* experiments using biomodels will enable quantitative evaluation of the effects of helical stents.

## Data Availability

The original contributions presented in the study are included in the article **Supplementary Material**, further inquiries can be directed to the corresponding authors.
