# Supplementary material for: Effects of helical centerline stent vs. straight stent placement on blood flow velocity
Source: Front Med Technol. 2023 Jun 2;5:1196125. doi: 10.3389/fmedt.2023.1196125 (PMC10272720; doi:10.3389/fmedt.2023.1196125)
Supplement: Supplementary file 1 [file Datasheet1.docx]

Supplementary Material

Effects of Helical Centerline Stent vs. Straight Stent Placement on Blood Flow Velocity

Yutaro Kohata, Makoto Ohta, Kazuyoshi Jin, Hitomi Anzai^*^

*** Correspondence:**Hitomi Anzai
hitomi.anzai.b5@tohoku.ac.jp

# Appearance of straight and helical stent

Supplementary Figure 1 shows the typical geometry of straight and helical stent. The detailed information of three commercial stent used was found in the link below.

ELUVIA (straight)…https://www.bostonscientific.com/en-US/products/stents--vascular/eluvia-drug-eluting-stent-system.html

LIFESTENT (straight)…https://www.bd.com/en-us/products-and-solutions/products/product-page.EX061203CS#overview

BioMimics 3D (helical)…https://veryanmed.com/technical-information/


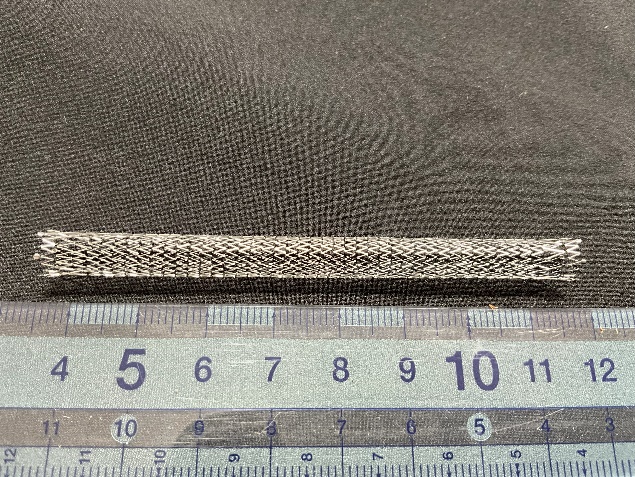

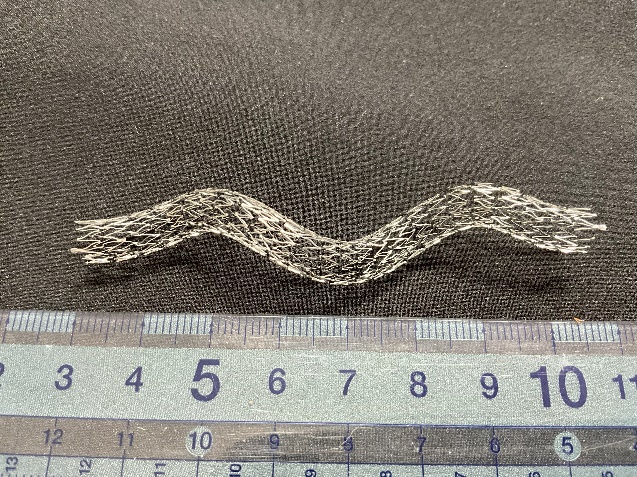


**Supplementary Figure 1.** Typical geometry of stents used. Left figure shows a straight-centerline stent (LifeStent™ Vascular and Biliary Stent, Becton Dickinson, USA) with a diameter of 6mm and length of 80mm. Right figure shows a helical-centerline stent (BioMimics3D, Veryan Medical, UK) with a diameter of 6mm and a length of 80mm.

# Setting for angiography images

Supplementary Table 1 summarized the condition for image acquisition of angiography.

**Supplementary Table 1. The angiography images acquired.**

| **Case** | **number of frames** | **fps (frame/s)** | **resolution (mm/px)** |
| --- | --- | --- | --- |
| 1 | 95 | 15 | 0.174 |
|  | 152 | 15 | 0.174 |
| 2 | 92 | 15 | 0.249 |
|  | 126 | 15 | 0.249 |
|  | 109 | 15 | 0.249 |
|  | 96 | 15 | 0.249 |
|  | 72 | 15 | 0.249 |
| 3 | 177 | 30 | 0.224 |
|  | 187 | 30 | 0.224 |
|  | 189 | 30 | 0.109 |
